# 15-deoxy-delta12,14-prostaglandin J2 attenuates endothelial-monocyte interaction: implication for inflammatory diseases

**DOI:** 10.1186/1476-9255-5-14

**Published:** 2008-08-08

**Authors:** Ratna Prasad, Shailendra Giri, Avtar K Singh, Inderjit Singh

**Affiliations:** 1 Department of Pediatrics, Medical University of South Carolina, Charleston, SC, 29425, USA; 2 Department of Pathology and Laboratory Medicine, Ralph Johnson Veterans Affairs Medical Center, Charleston, SC, 29425, USA

## Abstract

**Background:**

The Infiltration of leukocytes across the brain endothelium is a hallmark of various neuroinflammatory disorders. Under inflammatory conditions, there is increased expression of specific cell adhesion molecules (CAMs) on activated vascular endothelial cells which increases the adhesion and infiltration of leukocytes. TNFα is one of the major proinflammatory cytokines that causes endothelial dysfunction by various mechanisms including activation of transcription factor NF-κB, a key transcription factor that regulates expression of CAMs. Peroxisome proliferator-activated receptor gamma (PPARγ) is a member of the nuclear hormone superfamily of ligand-activated transcriptional factors. 15-deoxy-δ 12, 14-prostaglandin J2 (15d-PGJ2) is a well recognized natural ligand of PPARγ and possesses anti-inflammatory properties both *in vitro *and *in vivo*. This study aims to elucidate the mechanism of 15-PGJ2 on the adhesion of mononuclear cells to activated endothelial cells.

**Methods:**

To delineate the signaling pathway of 15d-PGJ2 mediated effects, we employed an *in vitro *adhesion assay model of endothelial-monocyte interaction. Expression of CAMs was examined using flow cytometry and real time PCR techniques. To define the mechanism of 15d-PGJ2, we explored the role of NF-κB by EMSA (Electrophoretic Mobility Shift Assay) gels, NF-κB reporter and p65-transcriptional activities by transient transfection in the brain-derived endothelial cell line (bEND.3).

**Results:**

Using an *in vitro *adhesion assay model, we demonstrate that 15d-PGJ2 inhibits TNFα induced monocyte adhesion to endothelial cells, which is mediated by downregulation of endothelial cell adhesion molecules in a PPARγ independent manner. 15d-PGJ2 modulated the adhesion process by inhibiting the TNFα induced IKK-NF-κB pathway as evident from EMSA, NF-κB reporter and p65 mediated transcriptional activity results in bEND.3 cells.

**Conclusion:**

These findings suggest that 15d-PGJ2 inhibits inflammation at multiple steps and thus is a potential therapeutic target for various inflammatory diseases.

## Background

Inflammatory mechanisms are pivotal in many disease states, including atherosclerosis, autoimmune disorders and ischemia/reperfusion injury [1-4]. Under inflammatory conditions there is activation of vascular endothelial cells that involves various morphological and metabolic changes [5]. There is induction of specific cell adhesion molecules, such as, intercellular adhesion molecule-1 (ICAM-1), vascular cell adhesion molecule-1 (VCAM-1) and E-selectin. These interact with their corresponding ligands on leukocytes namely, lymphocyte function-associated antigen-1 (LFA-1), very late antigen-4 (VLA-4) and carbohydrate moieties respectively [2,6]. The process of infiltration involves sequential capture, rolling, firm adhesion and transmigration across the endothelial barrier [7]. Blockade of CAMs that mediate the accumulation of mononuclear cells under inflammation is now considered as an effective treatment strategy in clinical inflammatory disorders.

TNFα is one of the major proinflammatory cytokines that is dysregulated in inflammatory diseases mentioned earlier and has been shown to contribute to endothelial dysfunction [8]. TNFα causes endothelial dysfunction by various mechanisms that includes activation of transcription factor NF-κB [9]. Transcriptional regulation of many pro-inflammatory genes, including CAMs, is under the control of different transcriptional factors including NF-κB [10,11]. NF-κB is a redox sensitive transcription factor that most commonly exists as a p50/p65 heterodimer. This heterodimer remains sequestered in the cytoplasm when associated with inhibitor of kappa B (IκB) proteins. Upon stimulation (e.g. by TNFα) IκB proteins get phosphorylated by upstream IκB kinases (IKKs) followed by degradation, releasing the active dimer to translocate into the nucleus to transcribe its target genes [12,13].

Peroxisome proliferator-activated receptors (PPARs) are members of the nuclear hormone superfamily of ligand-activated transcriptional factors. PPARs heterodimerize with retinoid × receptor (RXR) and bind to peroxisome proliferator-response elements in target genes [14]. The subtype PPARγ is a regulator of adipogenesis [15]. A number of studies have demonstrated that PPARγ may play a role in regulating inflammatory responses [16,17]. 15-deoxy-d 12, 14-prostaglandin J2, the ultimate metabolite of prostaglandin (PG) D_2_, is a natural ligand of PPARγ. 15d-PGJ2 has been shown to inhibit expression of iNOS and TNFα in several cell types that are dependent on PPARγ [18,19]. However, there are also anti-inflammatory responses of 15d-PGJ2 that are PPARγ independent [20,21]. There are studies that report protective effects mediated by 15d-PGJ2 via inhibition of infiltration of immune cells in various models of inflammation e.g. endotoxic shock [22], lung injury [23], ischemia/reperfusion injury [24] and experimental autoimmune encephalomyelitis (EAE) [25,26]. Thus, based on these studies, we hypothesized that 15d-PGJ2 inhibits the adhesion of mononuclear cells to the endothelial cells and thereby attenuates their transmigration. We observed that 15d-PGJ2 inhibited the adhesion of monocytes to bEND.3 endothelial cell line, activated by TNFα, by downregulation of endothelial CAMs via inhibition of IKK-NF-κB pathway.

## Methods

### Reagents and Antibodies

DMEM (4.5 g/L glucose), minimum essential medium alpha (MEM alpha) with ribonucleotides and deoxyribonucleotides, RPMI-1640 medium and FBS were purchased from Gibco BRL (Carlsbad, CA, USA). Granulocyte macrophage colony stimulating factor (GMCSF) and recombinant mouse TNFα were from R & D Systems (Minneapolis, MN, USA). Vybrant Cell adhesion kit containing Calcein AM fluorescent dye was from Molecular Probes (Eugene, OR, USA). ECL detection kit was from GE healthcare (Piscataway, NJ, USA). Antibodies for p65, p50, IκBα, VCAM-1 were purchased from Santa Cruz Biotechnologies (Santa Cruz, CA, USA). Texas red conjugated rabbit IgG antibody was from Vector Lab. Inc. (Burlington, CA, USA). Trizol reagent and Lipofectamine Plus were from Invitrogen (Carlsbad, CA, USA). Fluoromount-G was from Electron Microscopy Sciences (Hartfield, PA, USA). Antibodies against VCAM-1 (FITC labeled), ICAM-1 and E-selectin (PE labeled) were from BD Pharmingen (Franklin Lakes, NJ). Luciferase assay system was purchased from Promega (Madison, WI).

### Cell culture

The bEND.3 mouse brain endothelial cells were from ATCC (American Type Culture Collection, Manassas, VA, USA) and were cultured in Dulbecco's modified Eagle's medium (high glucose) supplemented with 10% Fetal Bovine serum (FBS) and antibiotics. Cells were grown to confluence, made serum free for further treatments, and stimulation with TNFα (50 ng/ml) for all the experiments. JAWS II, a mouse monocyte cell line (ATCC) was maintained in MEM Alpha medium with 10% heat inactivated FBS, 0.5% gentamycin and granulocyte-macrophage colony-stimulating factor (GMCSF) (1 ng/mL; R & D Systems).

### Plasmids and Transfection

NF-κB-luciferase was kindly provided by Dr. George Rewadi (Institut Pasteur, Laboratoire des Mycoplasmes, Paris, France), flag-IKKα was a gift from Dr. Zheng-Gang Liu (National Institute of Health, Bathesda, MD) and FLAG-tagged wild-type (wt) PPARγ and FLAG-tagged L468A/E471A PPARγ were provided by Dr. V. Chatterjee (University of Cambridge, Cambridge, U.K.). The peroxisome proliferator-response element (PPRE)-containing reporter plasmid (J6-thymidine kinase (TK)-Luc) was provided by B. Staels (Institut Pasteur de Lille, Lille, France). PTL-luciferase, Gal-p65 and Gal-DBD (DNA binding domain) were purchased from Panomics (Fremont, CA). The endothelial cell line was transfected with the indicated plasmid (0.5 μg/well) using Lipofectamine Plus Reagent under serum free conditions as described before [27]. pcDNA3.1 was used to normalize the total content of DNA in all transfection experiments.

### *In vitro *Adhesion assay model

As described earlier, bEND.3 cells were grown as monolayers in double chamber slides (Nalge Nunc, Naperville, IL, USA) [27]. Cells were pre-treated with 15d-PGJ2 for 30 min followed by TNFα for 6 h. Dye labeled monocytes at the concentration of 2 × 10^6 ^cells/ml were added per chamber on the bEND.3 cells and allowed to interact for 30 min with gentle shaking at 37°C. Adherent fluorescent cells were observed using a fluorescence microscope (Olympus, BX60) and images were captured in Adobe Photoshop 7.0 at 100×. Adherent fluorescent cells were counted using Image Pro-Plus 4.0 software. Mean and SD were calculated for independent experiments. Results were plotted as fold change compared to the control values for all the experiments.

### Immunocytochemistry

BEND.3 cells were grown in chamber slides and treated with 15d-PGJ2 and stimulated with TNFα for 20 min. Cells were fixed with paraformaldehyde (4%) followed by blocking in blocking reagent. Cells were then incubated in anti-p65 antibody followed by incubation in secondary antibody and mounting with Flouromount-G. The stained sections were analyzed by immunofluorescence microscopy (Olympus BX-60 from Opelco, Dulles, VA, USA) with images captured using an Olympus digital camera (Optronics, Goleta, CA, USA) at 400× magnification. Captured images were processed using Adobe Photoshop 7.0 and were adjusted using brightness and contrast tools. Three independent experiments were done and 5 fields for each treatment were taken. Representative images are shown.

### Real-time or quantitative (q) PCR

Cells were harvested in Trizol reagent and RNA was isolated per the manufacturer's protocol. cDNA synthesis was done using iScript CDNA synthesis kit (BIO-RAD Laboratories, Hercules, CA, USA) per the manufacturer's protocol. qPCR was performed using SYBR GREEN PCR master mix (Applied Biosciences, Foster city, CA, USA) and BIO-RAD laboratories iCycler iQ PCR using primers as described before [27]. primers of CAMs and 18S are as follows, ICAM-1 FP 5'-gca gag tgt aca gcc tct tt-3' RP 5'-ctg gta tcc cat cac ttg-3', VCAM-1 FP 5'-gca gag tgt aca gcc tct tt-3', RP 5'-ctg gta tcc cat cac tcg ag-3'; E-selectin FP 5'-act tca gtg tgg tcc aag ag-3' RP 5'-gca cat gag gac ttg tag gt-3'; 18S FP 5'-gaa aac att ctt ggc aaa tgc ttt-3' RP5'-gccgct aga ggt gaa att ctt-3'. The normalized mRNA expression was computed with that of 18s expression. Values are expressed as fold change from the control values and plotted.

### Preparation of cytosolic and nuclear extracts

Cytosolic and nuclear extracts from bEND.3 cells were prepared using the method of Digman et al [28] with slight modification [29]. Cells were harvested, washed twice with ice-cold PBS, and lysed in 400 μl of buffer A (10 mM HEPES, pH 7.9, 10 mM KCl, 2 mM MgCl_2_, 1 mM PMSF, 5 μg/ml aprotinin, 5 μg/ml pepstatin A, and 5 μg/ml leupeptin) containing 0.1% Nonidet P-40 for 15 min on ice, vortexed vigorously for 15 s, and centrifuged at 14,000 rpm for 30 s. The pelleted nuclei were resuspended in 40 μl of buffer B [20 mM HEPES, pH 7.9, 25% (v/v) glycerol, 0.42 M NaCl, 1.5 mM MgCl_2_, 0.2 mM EDTA, 1 mM PMSF, 5 μg/ml aprotinin, 5 μg/ml pepstatin A, and 5 μg/ml leupeptin]. After 30 min on ice, lysates were centrifuged at 14,000 rpm for 10 min. Supernatants containing the nuclear proteins were diluted with 20 μl of modified buffer C [20 mM HEPES, pH 7.9, 20% (v/v) glycerol, 0.05 M KCl, 0.2 mM EDTA and 0.5 mM PMSF] and stored at -70°C until use. Cytosolic fraction (50 μg) was used for western blot analysis for the detection of IκBα and IKKα using their specific antibodies as described before [29].

### Western blot

Cell extracts were prepared as previously described with lysis buffer (50 mM Tris-HCl, pH 7.4, containing 50 mM NaCl, 1 mM EDTA, 0.5 mM EGTA, 1% Triton X-100, 10% glycerol, and protease inhibitor mixture) [27,29]. Protein (50 μg) was loaded with appropriate marker (Bio-Rad Laboratories, Hercules, CA, USA) on 8% sodium dodecyl sulfate-polyacrylamide gel (SDS_.PAGE), followed by transfer to nitrocellulose membrane. The membrane was blocked with 5% milk or 3% BSA in Tris buffered saline-tween (TBST). Primary anti-p65, -IκBα, -pIKKα was added. Blots were washed, followed by incubation in secondary antibody and then detection by ECL-chemiluminescence method.

### Electrophoretic mobility shift assay (EMSA)

Nuclear extracts from treated and untreated cells were prepared and EMSA was performed as described previously [29,30] using NF-κB consensus sequence that was end-labeled with [γ-^32^P] ATP. Nuclear extracts were normalized on the basis of protein concentration and equal amounts of protein (5 μg) were loaded. The gels were dried and then autoradiographed at -70°C using x-ray film.

### Flow cytometry

15-PGJ2 treated and untreated bEND.3 cells in the presence or absence of TNFα (50 ng/ml) were harvested and processed as described earlier [31]. Cells were blocked with anti-CD16/CD32 and incubated with FITC- or PE-labeled antibodies against ICAM-1, VCAM-1 and E-selectin. The cells were acquired by FACS and analyzed by CellQuest (BD PharMingen, Franklin Lakes, NJ).

### Statistical analysis

Results shown represent mean ± SD. Statistical analysis was performed by ANOVA by the Student-Neumann-Keuls test using GraphPad InStat software (San Diego, CA, USA).

## Results

### 15d-PGJ2 inhibits monocyte adhesion to a brain-derived endothelial cell line

**Figure 1:**
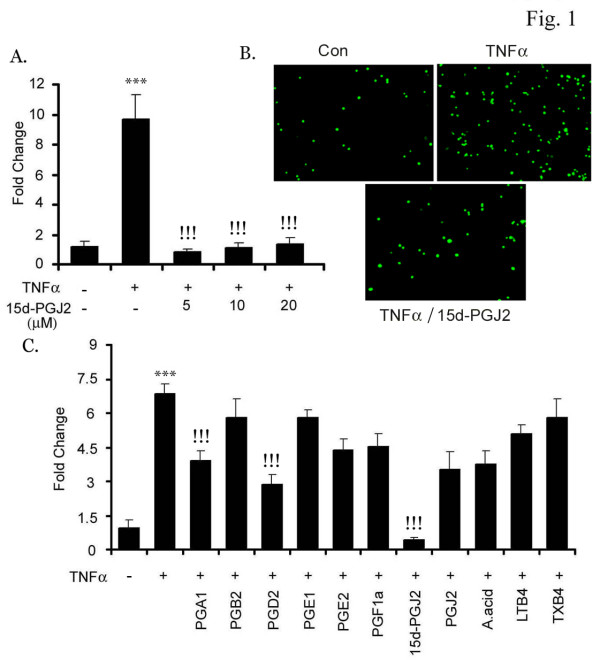
**5d-PGJ2 inhibits monocyte adhesion to endothelial cells**. bEND.3 cells were incubated with different concentrations of 15d-PGJ2 (1–20 μM) (A) or mentioned prostaglandins (5 μM), arachidonic acid (5 μM), Leukotriene 4 (LTB 4, 5 μM) and Thromboxanes 4 (TXB 4, 5 μM) (C) for 30 min followed by TNFα (50 ng/ml) stimulation for 6 h. Fluorescently labeled monocytes were allowed to interact with activated bEND.3 cells. Adhered monocytes were counted as mentioned in 'Material and Methods'. Data calculated as mean ± SD of 21 fields from 3 different experiments. *** p < 0.001 compared to untreated control cells and !!! p < 0.001 compared to TNFα treated cells. (B) is the pictorial representation of adhesion under TNFα (50 ng/ml) and 15d-PGJ2 (10 μM) treatment.

Activated bEND.3 endothelial cells under pro-inflammatory environment allows increased adherence of leukocytes to its surface to facilitate their migration [6]. In our *in vitro *system, bEND.3 cells were activated with TNFα that caused a significant increase in the adhesion of monocytes (~9 fold) compared to untreated cells. However, treatment with15d-PGJ2 (1–10 μM) 30 min prior to the addition of TNFα significantly inhibited the adhesion of monocytes (Fig. 1a, b). Prostaglandin production begins with the liberation of arachidonic acid which under cyclooxygenase enzymes 1 and 2 gets converted to PGH2. Specific prostaglandin synthase convert PGH2 into a series of prostaglandins including PGI2, PGF2α, PGD2 and PGE2 [32]. We also treated the bEND.3 cells with different prostaglandins (PGA1, PGB2, PGD2, PGE1, PGE2, PGF1α, 15d-PGJ2, PGJ2), arachidonic acid, leukotriene (LTB4) and thromboxane (TXB4) and observed that PGA1 and PGD2 treatment showed a significant decrease in TNFα induced adhesion of monocytes, as these are precursors of 15d-PGJ2 (Fig. 1c). These results suggest the specificity of 15d-PGJ2 in mediating the inhibition of the adhesion process of monocytes on activated bEND.3 cells. 15d-PGJ2 did not cause any cell death (assessed by MTT and LDH release assays) at the concentrations used (data not shown).

### 15d-PGJ2 inhibits expression of endothelial CAMs

**Figure 2:**
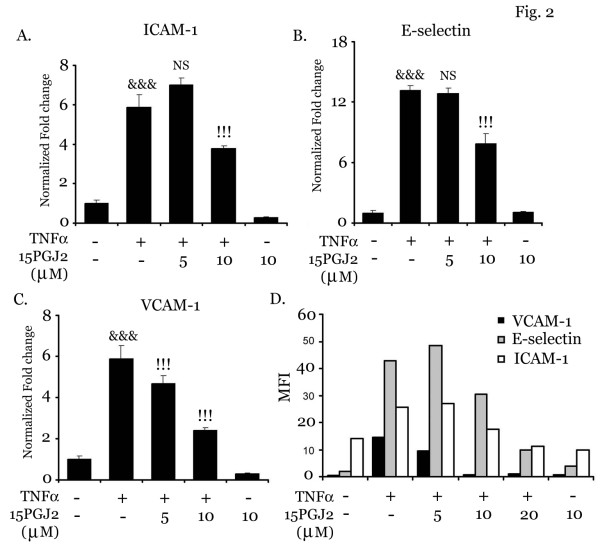
**15d-PGJ2 inhibits mRNA and protein expression of endothelial CAMs**. bEND.3 cells were pretreated with 15d-PGJ2 (5–20 μM) for 30 min followed by stimulation with TNFα (50 ng/ml) for 2 h. Cells were harvested in Trizol reagent for RNA isolation and cDNA synthesis. RT-PCR analysis was done for ICAM-1 (A), VCAM-1 (B) and E-selectin (C). Results were calculated as mean ± SD for 3 independent experiments. Samples were examined in triplicates. &&& p < 0.001 compared with control (untreated and unstimulated cells) and !!! p < 0.001 as compared to TNFα treatment. For the quantitation of expression of surface CAMs, bEND.3 cells were treated with TNFα (50 ng/ml) in the presence or absence of 15d-PGJ2 (5–20 μM) for 6 h followed by flow cytometry analysis (D) (n = 2).

Extravasation of mononuclear cells the recruitment cascade are orchestrated by cell adhesion molecules on both endothelial and immune cells [1]. Accordingly, we examined the effect of 15d-PGJ2 on TNFα induced expression of CAMs (VCAM-1, ICAM-1 and E-selectin). For this, bEND.3 cells were pretreated with15d-PGJ2 (5–10 μM) followed by TNFα (50 ng/ml) treatment. After 2 h of incubation, bEND.3 cells were processed for RNA isolation and quantitative analysis of CAMs using real time PCR (qPCR). Treatment with TNFα significantly increased the mRNA expression of VCAM-1, ICAM-1 and E-selectin as compared to control cells. 15d-PGJ2 markedly downregulated their expression with a most pronounced effect observed on expression of VCAM-1 as compared to E-selectin or ICAM-1 (Fig. 2a, b and 2c). These observations are in agreement with flow cytometry analysis which also showed that 15d-PGJ2 treatment significantly reduced the expression of endothelial CAMs with maximum affect on VCAM-1 expression (Fig. 2d).

### 15d-PGJ2 inhibits VCAM-1 expression in a PPARγ independent manner

**Figure 3:**
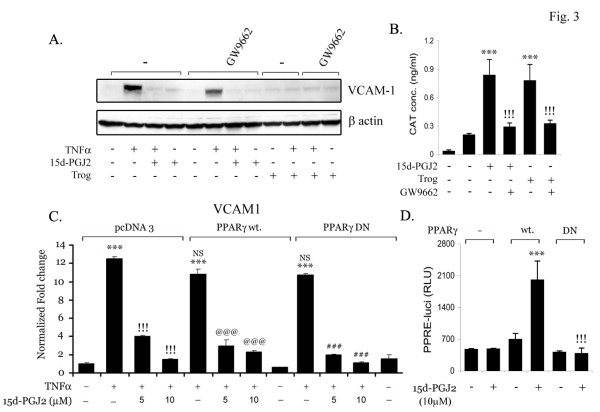
**15d-PGJ2 inhibits VCAM-1 in PPARγ independent manner**. bEND.3 cells were treated with GW9662 (10 μM) 30 min prior to treatment with 15d-PGJ2 (10 μM) or troglitazone (10 μM) followed by TNFα treatment (50 ng/ml). bEND.3 cells were lysed and processed for immunoblot analysis for VCAM-1 and β actin expression (A). Endothelial cell line was cotransfected with PPARγ-GAL4 chimeras and the reporter plasmid (upstream activating sequences)_5_-TK-CAT. After 48 h, cells were treated with 15d-PGJ_2 _or trogliatzone in the presence or absence of GW9662 (10 μM) for 24 h. Cell extracts were subsequently assayed for CAT activity by ELISA (Roche) (B). pCMV-GAL4-binding domain (without insert) and (upstream activating sequences)_5_-TK-CAT were transfected as a control to detect the basal levels of CAT activity (first lane). Data are mean of three values ± SD. *** *p *< 0.001 as compared with untreated cells; !!! *p *< 0.001 as compared with 15d-PGJ2 treated cells. (C) Cells were transfected with PPARγ wild type (Wt) and dominant negative (DN) constructs followed by treatment with 15-dPGJ2 (5 and 10 μM; 30 min) and TNFα (50 ng/ml, 2 h) and processed for qPCR for detection of VCAM1 mRNA expression as described in 'Material and Methods' (C). Results were calculated as mean ± SD for 3 independent experiments. Samples were run in triplicates. &&& p < 0.001 compared with control (untreated and unstimulated cells) and !!! p < 0.001 as compared to TNFα treatment. Cells were co-transfected with PPARγ wild type (Wt) and dominant negative (DN) (0.5 μg/well) constructs along with PPRE-luc reporter (0.5 μg/well) and pRL-TK (0.5 μg/well) followed by treatment with 15-dPGJ2 (10 μM) after 24 h. After 24 h incubation, luciferase activity was performed, as described before pcDNA3.1 was added to normalize the total content of DNA for transfection. Data are mean ± SD of three different values. ***, *p *< 0.001 as compared with untreated cells; !!!, *p *< 0.001 as compared with 15d-PGJ_2_-treated PPAR wt transfected cells.

To determine whether 15d-PGJ_2 _mediates its inhibitory effect through PPARγ, we employed GW9662, an irreversible PPARγ antagonist. GW9662 (10 μM) did not reverse 15d-PGJ2 mediated inhibition of TNFα induced expression of VCAM-1 in the endothelial cell line (Fig. 3A). Another activator of PPARγ, troglitazone, was used to examine if PPARγ plays any role in expression of VCAM-1 in bEND.3 cells. Troglitazone treatment, similar to 15d-PGJ2 treatment, inhibited the expression of VCAM-1, which could not be reversed by GW9662 (Fig. 3a). To examine the ability of GW9662 on 15d-PGJ2 and troglitazone mediated induction of PPARγ transcription, we used a chimeric receptor system in which the putative ligand-binding domain of the PPARγ is fused to the DNA binding domain of the yeast transcription factor galactose-responsive gene 4 (GAL4). The 15d-PGJ_2 _and troglitazone potently activated the PPARγ-dependent chloramphenicol acetyltransferase (CAT) reporter activity, which was completely blocked by GW9662 treatment (Fig. 3b). To confirm this observation, bEND.3 cells were transfected with PPARγ wild type (Wt) and dominant-negative (DN) expression vectors and determined the effects on VCAM-1mRNA expression. 15d-PGJ2 was able to inhibit the TNFα induced expression of VCAM-1 in both control and PPARγ Wt transfected cells. However, transfection with PPARγ DN was not able to attenuate the 15-dPGJ2 mediated inhibition of VCAM-1 mRNA expression indicating that the inhibitory effect of 15d-PGJ2 is independent of PPARγ (Fig. 3c). Treatment with 15d-PGJ2 induced the PPRE-luciferase activity in transiently transfected PPARγ Wt expression vector, whereas, it had no effect in PPARγ DN transfected cells (Fig. 3d) suggesting that 15d-PGJ2 has the ability to activate PPARγ but its effect on VCAM-1 expression in the bEND.3 endothelial cell line is independent of PPARγ.

### 15d-PGJ2 inhibits NF-κB function in brain-derived endothelial cell line

**Figure 4:**
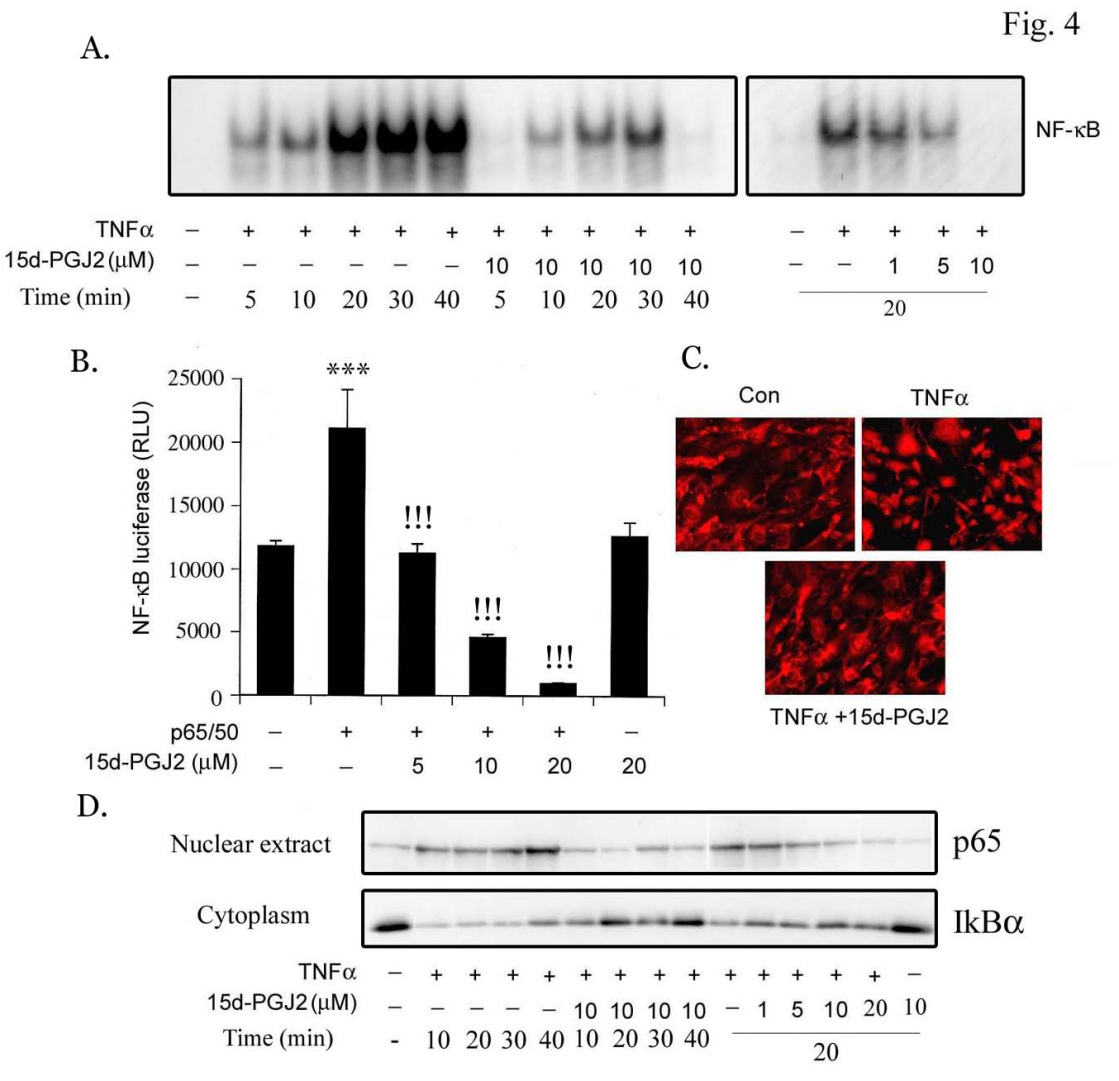
**15d-PGJ2 inhibits TNFα induced NF-κB function in endothelial cells**. bEND.3 cells were treated with 15d-PGJ2 (1–10 μM) and TNFα (50 ng/ml) for various time periods (5–40 min) and processed for EMSA as described in 'Material and Methods' (A). bEND.3 cells were transiently transfected with p65, p50 expression vectors along with NF-κB luciferase reporter construct (0.5 μg/well) and pCMV-β-galactosidase (0.5 μg/well) followed by treatment with 15d-PGJ2 (5–20 μM) for 4 h and processed for luciferase and β-galactosidase activities. Luciferase activity was normalized with respect to β-gal activity (B). Results were calculated as mean ± SD for 3 independent experiments. Samples were run in triplicates. &&& p < 0.001 compared with control and !!! p < 0.001 compared with TNFα treatment (50 ng/ml). Cells were treated with 15d-PGJ2(10 μM) for 30 min followed by TNFα for 20 min and stained with anti-p65 antibody as described in 'Material and Methods' (C). Images taken at 200× magnification are representative of 6 fields from each treatment and 3 independent experiments. Treated and untreated cells were processed for immunoblot analysis for p65 and IκBα levels (D). Representative blot from two independent experiments are shown.

To further understand the mechanism of inhibitory action of 15d-PGJ2 on endothelial CAMs and the process of adhesion we examined the effect of 15d-PGJ2 on NF-κB pathway, which is a pleiotropic regulator of many genes involved in inflammation including CAMs [11]. Using EMSA, we observed that 15d-PGJ2 inhibited the TNFα induced binding of the NF-κB complex, in a time and dose-dependent manner (Fig. 4a). To further define the inhibitory effect of 15d-PGJ2 on TNFα mediated activation of the NF-κB pathway, the bEND.3 cells were transfected with the p65/p50 complex along with the NF-κB luciferase reporter construct. Cells transfected with p65/p50 exhibited increased reporter activity, which was markedly reduced in a dose-dependent manner with 15d-PGJ2 treatment (Fig. 4b). These observations obtained from EMSA and transfection studies were further confirmed by immunostaining for p65 nuclear translocation. Under TNFα stimulation, p65 translocated to the nucleus and was markedly attenuated by 15d-PGJ2 treatment (Fig. 4c). Correspondingly, we also observed that 15d-PGJ2 inhibited the TNFα induced nuclear translocation of p65 and degradation of IκBα protein in a time and dose-depended manner (Fig. 4d).

**Figure 5:**
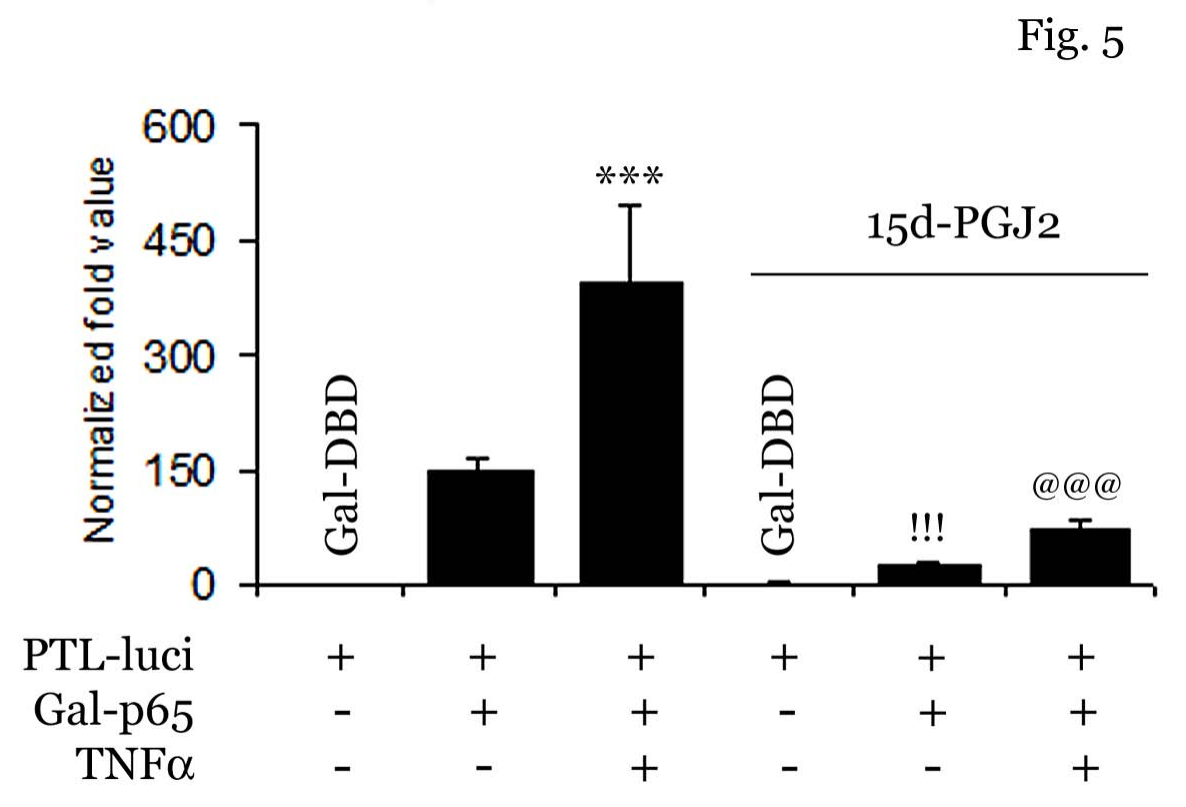
**15d-PGJ2 inhibits p65 transcriptional activity in endothelial cells**. bEND.3 cells were transfected with Gal-p65 or Gal-DBD along with PTL-luciferase and PRL-TK reporter constructs as described in Material and Method. bEND.3 cells were pretreated with 15d-PGJ2 (10 μM) for 30 min followed by TNFα treatment (50 ng/ml). After 6 h of TNFα treatment, cells were processed for luciferase assay and results were normalized with PRL-TK luciferase activity in each sample. Results were calculated as mean ± SD for 3 independent experiments. *** and !!! p < 0.001 compared with control, @@@ p < 0.001 compared with TNFα treatment.

To support the 15d-PGJ2 mediated inhibition on NF-κB pathway, we examined the effect of 15d-PGJ2 on p65-DNA binding domain-gal4 transcriptional activity. The p65-DNA binding domain-gal4 (p65-DNA-gal4) is a chimeric-transactivator, which consists of transcriptional activation domain of NF-κB p65 protein fused to the DNA-binding domain of GAL4 protein from yeast. As evident from figure 5, treatment with TNFα induced the transcriptional activity of p65-DBD-gal4 which was completely blocked by 15d-PGJ2 treatment.

### Inhibition of IKK activity by 15d-PGJ2

**Figure 6:**
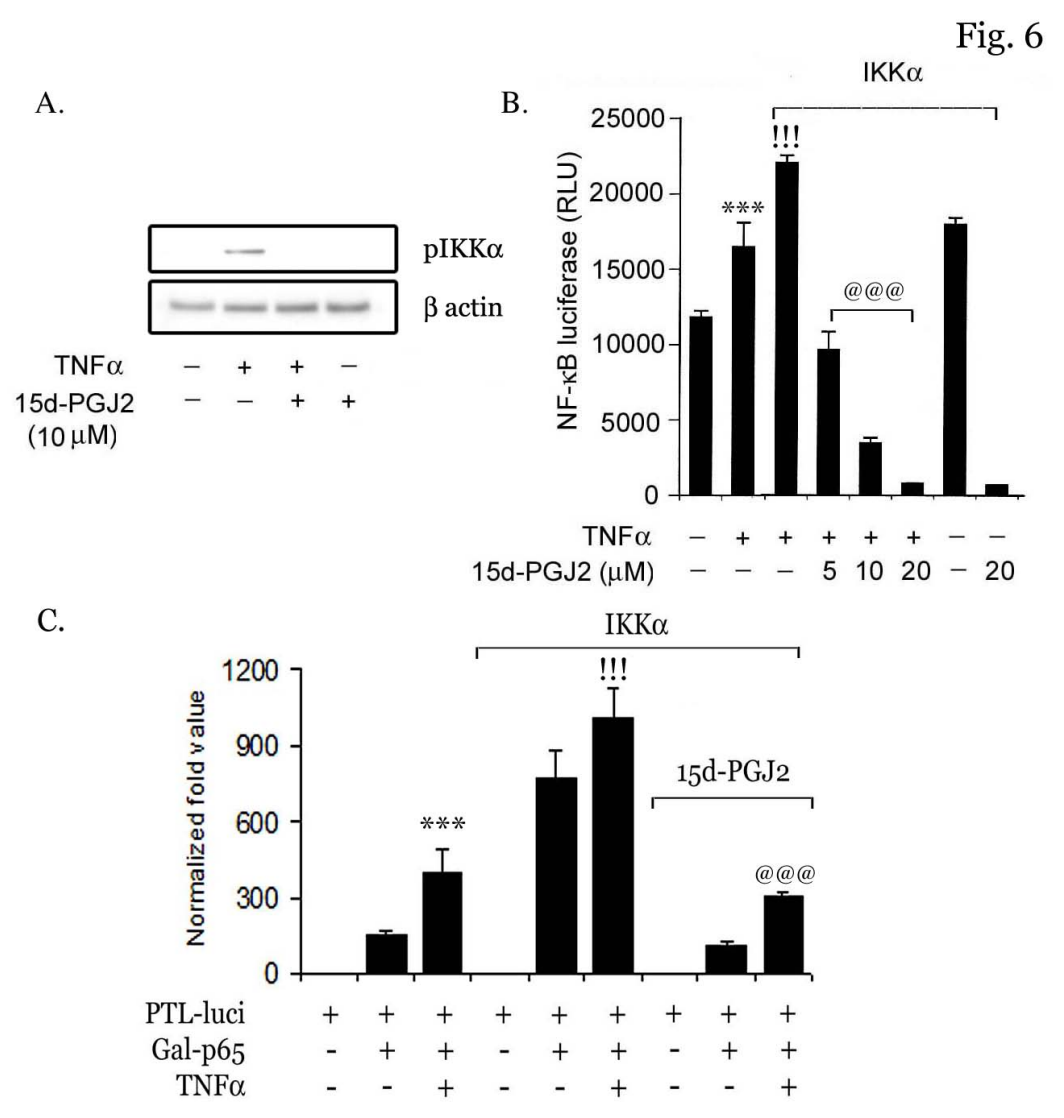
**15d-PGJ2 inhibits TNFα induced IKKα mediated NF-κB reporter activity**. bEND.3 cells were treated with TNFα (50 ng/ml) in the presence or absence of 15d-PGJ2 (10 μM) followed by detection of pIKKα using its specific antibody (Cell Signaling) (A). β actin was used as a control for equal content of protein loaded. bEND.3 cells were transfected with IKKα, NF-κB luciferase and pCMV-β-galactosidase constructs and treated with 15d-PGJ2 (5–20 μM) and TNFα (50 ng/ml). After 4 h of TNFα treatment, cells were processed for luciferase assay as described in 'Material and Methods' (B). Results were calculated as mean ± SD for 3 independent experiments. &&& p < 0.001 compared with control, !!! p < 0.001 compared with TNFα treatment and ### p > 0.001 compared with IKKα. bEND.3 cells were transfected with Gal-p65 or Gal-DBD in the presence or absence of flag-IKKα along with PTL-luciferase and PRL-TK reporter constructs as described in Material and Method. bEND.3 cells were pretreated with 15d-PGJ2 (10 μM) for 30 min followed by TNFα treatment. After 6 h of TNFα treatment (50 ng/ml), cells were processed for luciferase assay and results were normalized with PRL-TK luciferase activity in each sample (C). Total DNA content was normalized with pcDNA3. Results were calculated as mean ± SD for 3 independent experiments.

Based on preceeding results, we examined the effect of 15d-PGJ2 on the activity of IKK, the upstream kinase of the NF-κB pathway. Cells were treated with 15d-PGJ2 followed by TNFα for 15 min and phosphorylation of IKKα was detected using a specific antibody. As shown in figure 6a, TNFα treatment induced phosphorylation of IKKα in bEND.3 cells which was completely blocked by 15d-PGJ2 treatment. bEND.3 cells were further cotransiently transfected with IKKα and NF-κB luciferase reporter constructs and after 24 h, cells were treated with TNFα with or without 15d-PGJ2. TNFα induced the IKKα mediated NF-κB-reporter activity, which was a significantly downregulated by 15d-PGJ2 treatment (Fig. 6b). This observation was further supported when bEND.3 cells were transiently cotransfected with p65-DBD-gal4 and IKKα expression vectors. Transient transfection with IKKα significantly induced p65 transcriptional activity which was completely blocked by 15d-PGJ2 treatment (Fig. 6c) suggesting that 15d-PGJ2 inhibits NF-κB function by inhibiting IKKα activity in bEND.3 cells.

### Post treatment of 15d-PGJ2 inhibits adhesion of monocytes on activated brain-derived endothelial cell line

**Figure 7:**
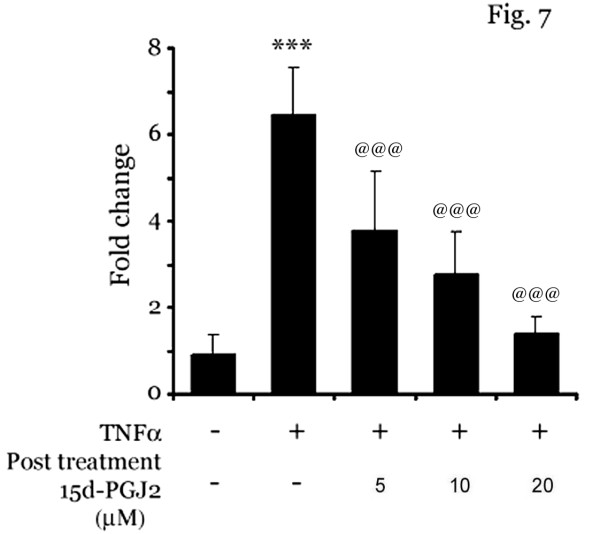
**Post treatment of 15d-PGJ2 inhibits monocyte adhesion to activated endothelial cells**. bEND.3 cells were treated with TNFα (50 ng/ml) for 6 h, followed by addition of different concentrations of 15d-PGJ2 (5–20 μM). After 30 min of incubation with 15-PGJ2, fluorescently labeled monocytes were allowed to interact with activated bEND.3 cells. Adhered monocytes were counted as mentioned in 'Material and Methods'. Data calculated as mean ± SD of 21 fields from 3 different experiments. *** p < 0.001 compared to untreated control cells and @ p < 0.001 compared to TNFα treated cells.

Our results suggested that 15d-PGJ2 inhibits the adhesion of mononuclear cells on activated endothelial cells by inhibiting the CAMs expression via downregulation of NF-κB pathway when pretreated before stimulation with TNFα. We wanted to examine if post treatment with 15d-PGJ2 could inhibit the adhesion of mononuclear cells on TNFα-stimulated cells. For this, bEND.3 cells were stimulated with TNFα for 6 h followed by addition of various concentrations (5–20 μM) of15d-PGJ2. After 30 min of treatment with 15d-PGJ2, cells were washed and labeled monocytes were added for adhesion assay. Interestingly, post treatment with 15d-PGJ2 inhibited adhesion of mononuclear cells on activated bEND.3 cells (Fig. 7) suggesting that 15d-PGJ2 probably inhibits multiple pathways including NF-κB-CAMs expression and other signaling pathway required for monocyte-endothelial cell adhesion,.

## Discussion

PGs are small lipid molecules that regulate numerous processes in the body and their biological effects is an area of concentrated research [33]. The J series of PGs have been demonstrated to regulate processes like adipogenesis, inflammation and tumorigenesis [32]. 15d-PGJ2 is a metabolite of PGD2 and is produced by mast cells, T cells, platelets and alveolar macrophages [34]. 15d-PGJ2 is emerging as a key anti-inflammatory mediator. Consistent with this we have previously shown that 15d-PGJ2 has an anti-inflammatory role in primary astrocytes [29]. This study reports for the first time that 15d-PGJ2 inhibits adhesion of monocytes to TNFα activated bEND.3 endothelial cells by downregulating endothelial CAMs via inhibition of IKKα-NF-κB pathway but in a PPARγ independent manner.

Infiltration of leukocytes is a crucial response in inflammatory reactions in numerous disorders where these leukocytes are intended to induce inflammation in CNS when BBB is compromised. However, when misdirected, they destroy healthy cells and matrix components causing tissue damage [1]. Therefore, in recent years efforts have been directed to limit the infiltration of mononuclear cells so as to minimize the tissue injury during the disease process. In earlier studies in different disease models, it was reported that 15d-PGJ2 inhibits infiltration of leuckocytes to site of inflammation [29,35]. Since adhesion of infiltrating cells to endothelium, is a prerequisite for infiltration, we investigated the effect of PPAR activator 15d-PGJ2 on the adhesion process. 15d-PGJ2 was observed to inhibit the adhesion of monocytes to activated bEND.3 endothelial cells in a dose-dependent manner. These Results were consistent with previous studies where 15d-PGJ2 inhibited the adhesion of mononuclear cells to PMA, IFNγ or IL-1β activated endothelial cells [36,37]. The inhibition of the adhesion process by15d-PGJ2 was mediated by down regulation of TNFα induced endothelial CAMs expression, namely, VCAM-1, E-selectin and ICAM-1. Further, this effect was found to be PPARγ independent. Our Results were consistent with other reports in which 15d-PGJ2 and other PPAR activators negatively modulate endothelial CAMs *in vitro *[37-39]. Treatment of bEND.3 cells with 15d-PGJ2 showed effects by attenuating signaling taking place during adhesion process as well as downregulating endothelial CAMs expression, thereby giving a significant additive effect on inhibition on adhesion of monocytes. To further understand the mechanism of inhibition mediated by15d-PGJ2, we determined its effect on the NF-κB transcription factor which is known to be activated by TNFα [9]. 15d-PGJ2 was observed to inhibit DNA binding of the NF-κB complex in a gel shift assay. Interestingly, this inhibition was through modulation of upstream targets of the NF-κB pathway. There was inhibition of TNFα induced degradation of IkBα protein thereby preventing p65 nuclear translocation. Our study is supported by other reports of inhibition of NF-κB by 15d-PGJ2, though in different cell types [29,35,40,41]. Thus, our data showed that 15d-PGJ2 inhibits TNFα induced NF-κB activity and consequently the expression of endothelial CAMs under our experimental model. Moreover, we have previously suggested IKK as a target of 15d-PGJ2 in modulating NF-κB pathway in brain glial cells [29,35], which is consistent in endothelial cells too. We can conclude from our *in vitro *data that 15d-PGJ2 inhibits endothelial-monocyte interactions via IKK-NF-κB-CAMs pathway in endothelial cells. PI3 kinase and Akt are also known to play an important role in the adhesion process [42]. The activation of IKK is also regulated via phosphorylation by Akt [43]. 15d-PGJ2 has been demonstrated to inhibit the PI3 kinase/Akt pathway in brain glial cells [29]. PI3 kinase and Akt pathway play important role in adhesion as we have documented before that inhibition of PI3Kinase and Akt is able to inhibit the adhesion of monocytes [27].

Thus, 15d-PGJ2 might be modulating PI3 kinase-Akt-IKK-NF-κB-CAMs pathway. Interestingly, post treatment with 15d-PGJ2 was also able to inhibit monocyte adhesion on activated bEND.3 cells, suggesting the possibility that15d-PGJ2 may also inhibit other signaling pathway/s important for firm and sustained adhesion of monocyte on endothelial cells.

15d-PGJ2 is a natural ligand of PPARγ and has numerous effects which are PPARγ dependent. Moreover, it has been shown to has therapeutic potential in various human autoimmune diseases as well as animal models of autoimmunity, including arthritis [44-46], ischemia-reperfusion injury [47,48], Alzheimer's disease [49-51], lupus nephritis [52,53] and EAE [26,54,55]. More recent evidences have shown that there are effects of 15d-PGJ2 that are independent of PPARγ activation [32], while, the exact mechanism of action of 15d-PGJ2 in different systems is unknown. There are various propositions such as presence of another cytoplasmic PG receptor [56], recruitment of p300 by NF-κB [29], inhibition of NF-κB DNA binding by alkylation of cysteine residue of p65 [57], or ROR dependent mechanism [39].

## Conclusion

All together, the present data shows that 15d-PGJ2 regulates inflammatory responses by inhibiting the infiltration of leukocytes across the endothelial barrier, which it does so by inhibiting monocyte adhesion to activated endothelial cells via downregulation of IKK-NF-κB-CAMs pathway in endothelial cells, independent of PPARγ.

## Abbreviations

15d-PGJ 2: 15-deoxy-Delta (12, 14)-prostaglandin J; CAM: cell adhesion molecule; ICAM: Intercellular cell adhesion molecule-1; VCAM-1: Vascular cell adhesion molecule-1; NF-κB: Nuclear factor kappa B; IκB: Inhibitory kappa B; IKK: Inhibitory kappa B kinase.

## Competing interests

The authors declare that they have no competing interests.

## Authors' contributions

This study is based on an original idea of SG and IS. RP and SG wrote the manuscript. SG directed and RP performed the *in vitro *experiments. AKS helped in finalizing manuscript. All authors read and approved the final manuscript.
